# PPAR-alpha and PPAR-beta expression changes in the hippocampus of rats undergoing global cerebral ischemia/reperfusion due to PPAR-gamma status

**DOI:** 10.1186/1744-9081-10-21

**Published:** 2014-06-16

**Authors:** Ying Luo, Qin He, Ge Kuang, Qingsong Jiang, Junqing Yang

**Affiliations:** 1 Department of Pharmacology, Chongqing Key Laboratory of Biochemistry and Molecular Pharmacology, Chongqing Medical University, Medical College Rd. No 1, Chongqing 400016, P. R. China; 2 The First Affiliated Hospital, Chongqing Medical University, Chongqing 400016, P. R. China

**Keywords:** Global cerebral ischemia-reperfusion injury, Hippocampus, Peroxisome proliferator-activated receptors

## Abstract

**Background:**

Peroxisome proliferator-activated receptors (PPARs, including alpha, beta and gamma subtypes) and their agonists have a protective role in treatment of central nervous system (CNS) diseases. The present study was designed to investigate the expression changes of PPAR-alpha, -beta, -gamma and NF-kappa B in the hippocampus of rats with global cerebral ischemia/reperfusion injury (GCIRI) after treatment with agonists or antagonists of PPAR-gamma.

**Methods:**

A rat GCIRI model was established by occlusion of bilateral common carotid arteries and cervical vena retransfusion. GW9662 (5 μg), a selective PPAR- gamma antagonist, was intraventricularly injected at 0.5 h before GCIR; Rosiglitazone (0.8, 2.4 and 7.2 mg/kg), a selective PPAR- gamma agonist, was injected intraperitoneally at 1 h before GCIRI. The expression changes of PPAR-alpha, -beta and -gamma at mRNA and protein levels were detected by RT-PCR and western blotting. The changes of spatial learning and memory (SLM) functions were assessed by using a Morris water maze; the pathohistological changes of hippocampal neurons were evaluated by hematoxylin-eosin (HE) staining; the contents of IL-1, IL-6, IL-10 and TNF-alpha, and the NF- kappa B expression were measured by enzyme-linked immunosorbent assay (ELISA) and immunohistochemical staining. The superoxide dismutase (SOD) activity and malondialdehyde (MDA) content were also detected.

**Results:**

The SLM function and hippocampal neurons were significantly impaired after the occurrence of GCIRI. The MDA, IL-1, IL-6, IL-10, TNF-alpha content and expression of PPARs increased significantly, but the SOD activity and NF-kappa B expression were weakened in the hippocampus. Rosiglitazone treatment significantly protected rats from SLM function impairment and neuron death, and resulted in higher expressions of SOD activity and NF-kappa B, but lower contents of MDA and inflammatory factors. After treatment with rosiglitazone or GW9662, no significant change in PPAR-alpha or -beta expression was detected.

**Conclusions:**

Rosiglitazone, a PPAR-gamma agonist, plays a protective role in hippocampal neuron damage of GCIRI rats by inhibiting the oxidative stress response and inflammation. The activation or antagonism of PPAR-gamma did not affect the expression of PPAR-alpha or -beta, indicating that the three subtypes of PPARs act in independent pathways in the CNS.

## Background

Peroxisome proliferator-activated receptors (PPARs), which belong to the nuclear receptor family of ligand-activated transcription factors, were originally described as gene regulators of various metabolic pathways, such as metabolism, adipogenesis, trophoblast differentiation, cell migration and inflammation control [1]–[6]. PPAR-α is mainly expressed in brown adipose tissues, the liver, muscles and the kidney; it is mainly involved in regulating lipid metabolism, insulin sensitivity and glucose homeostasis [7,8]. PPAR-β is expressed all over the body and participates in embryonic development, implantation, bone formation and lipid metabolism [9,10]. PPAR-γ is mainly expressed in adipose tissues, colonic epithelia, macrophages, the liver, the spleen and the kidney; it plays an important role in insulin sensitivity, cell cycle regulation and cell differentiation [11]. In the past decade, tremendous progress has been made towards understanding the physiological roles of PPARs in the occurrence and development of many human diseases, including diabetes, obesity, atherosclerosis, hypertension and cancer.

Global cerebral ischemia/reperfusion injury (GCIRI) occurs in patients who are successfully resuscitated from various clinical conditions such as cardiac arrest, asphyxia and shock, which are frequently accompanied by inflammation and can lead to serious neuronal injury, and further to neurodegeneration and learning and memory impairment [12]. Proinflammatory cytokines, such as interleukin (IL)-1β, IL-6 and tissue necrosis factor (TNF)-α, have been implicated as important mediators of injury following cerebral ischemia [13] and contribute to pathogenesis, exacerbating brain tissue damage following ischemia/reperfusion (I/R) injury [14].

In addition to regulating metabolism, activation of PPARs results in anti-inflammation and antioxidative effects [15]. Intriguingly, recent reports show that activation of PPARs is helpful in regulating neuronal death in patients with ischemic brain injury and neurodegenerative diseases [16]–[19]. The expression level of PPAR-α genes in the hippocampus and the improvement of cognitive performance were increased by the reduction of the n-6:n-3 fatty acid ratio [20]. The effects of palmitoylethanolamide (PEA) on astrocyte activation and neuronal loss and subsequently the improved neuronal survival in models of amyloid-β (Aβ) neurotoxicity are dependent on the expression of PPAR-α [21]. Genetic ablation of PPAR-α in mice exacerbated the systemic toxicity of 1-methy l-4 -phenyl-1,2,3,6-tetrahyropyridine (MPTP), while PEA-induced neuroprotection was partially PPAR-α-dependent [22]. The central administration of PPAR-δ/β agonists significantly and dose-dependently attenuated the ischemic brain damage after reperfusion in rats [23]. GW0742 as an agonist of PPAR-δ/β exerts significant neuroprotective effects in rats with GCIRI via PPAR-δ/β activation and its anti-inflammation effect [24].

PPAR-γ is the focus among the three PPAR subtypes in terms of their neuroprotective effects. In animal models of neurological and cardiovascular diseases, rosiglitazone prevents neuronal cell death and reduced infarct volume after ischemia and reperfusion [25]. Thiazolidinediones (an agonists of PPAR-γ) modulate the maturation and differentiation of microglia and astrocytes, and inhibit the production of nitric oxide (NO), pro-inflammatory cytokines TNF-α, IL-1β and IL-6, and chemokine MCP-1 from microglia and astrocytes [26]. In addition, pioglitazone increases the cerebral level of Cu-Zn superoxide dismutase (Cu-Zn SOD) and significantly reduces the infarct size induced by transient but not permanent MCAO [27]. Intraventricular administration of pioglitazone 24 h before MCAO significantly reduces the expressions of IL-1, COX-2 and inducible nitric-oxide synthase (iNOS) induced by inflammation [28]. These results demonstrate that PPAR-γ agonists have protective effects on focal cerebral ischaemia-reperfusion injury (IRI), and that the action mechanisms include inhibition of inflammatory reaction and oxidative stress. However, those studies focus on the neuroprotective effect of PPAR-γ in focal ischemia models, but not on the expression and effect of PPAR-γ in the GCIRI model.

Four major domains have been identified in PPARs: A/B, C, D and E/F. Domain C is comprised of about 70 amino acids and encodes the DNA-binding domain (DBD). Domain E, which is the ligand-binding domain (LBD), is responsible for ligand specificity and activation of PPAR binding to the peroxisome proliferator response element (PPRE) with resultant modulation of gene expression. In humans, there is about an 86% identity between PPAR-α and -β and 83% identity between PPAR-α and -γ1 in DBD, and about a 70% identity between PPAR-α and -β and 68% identity between PPAR-α and -γ1 in LBD [10], which are similar in humans. However, the sequences of PPARs have high identity in rats: about an 86% identity between PPAR-α and -β and 83% identity between PPAR-α and -γ in DBD, and about a 66% identity between PPAR-α and -β and 62% identity between PPAR-α and -γ in LBD. Under such percentages of identity in the structure among those three isotypes, it has not been characterized whether the expression change of PPAR-γ will affect those of the other subtypes, or whether activation or inhibiton of PPAR-γ will affect the expressions of the other subtypes.

The objectives of the present study are to investigate whether the PPAR-γ agonist exerts beneficial action on the memory and learning function by anti-oxidant and inhibits inflammatory reaction: investigate the expression of PPARs in the hippocampus of GCIRI rats, to evaluate the changes of PPAR-α and -β expressions due to agonism or antagonism of PPAR-γ, and to test whether the three PPAR isotypes are acting in independent pathways in the CNS. The results will provide a basis for combination of PPARs to prevent GCIRI in the clinical setting.

## Method

### Animals and experimental design

Experiments were approved by the Animal Laboratory Administrative Center and the Institutional Ethics Committee at Chongqing Medical University. 84 Sprague–Dawley (SD) male rats (200–250 g, from the Laboratory Animal Center of Chongqing Medical University) were subjected to bilateral carotid artery occlusion. Prior to the artery occlusion, the rats were randomly allocated to the following groups:

1. Sham group: The animals were subjected to the same surgical procedures as other groups but the common carotid arteries were not occluded (n = 12).

2. Ischemia/Reperfusion (I/R) group: The rats received GCIR (n = 12).

3. 0.8 mg/kg rosiglitazone group: Identical to ischemia model group, except for receiving rosiglitazone (a kind gift from Prof. Xi-He Yan from Department of Medicinal Chemistry of Chongqing Medical University) 0.8 mg/kg i.p. 1 hour prior to artery occlusion (n = 12).

4. 2.4 mg/kg rosiglitazone group: Identical to ischemia model group except for receiving rosiglitazone 2.4 mg/kg i.p. at 1 hour prior to artery occlusion (n = 12).

5. 7.2 mg/kg rosiglitazone group: Identical to ischemia model group except for receiving rosiglitazone 7.2 mg/kg i.p. at 1 hour prior to artery occlusion (n = 12).

6. 7.2 mg/kg rosiglitazone plus GW9662 group: Identical to ischemia model group except for receiving GW9662 (Alexis, USA) 5 μg, i.c.v. [29], at 30 min prior to artery occlusion and rosiglitazone 7.2 mg/kg i.p. at 1 hour prior to artery occlusion (n = 12).

7. GW9662 in sham control group: Identical to sham group except for receiving GW9662 5 μg, i.c.v., at 30 min prior to sham surgery (n = 12).

### GCIRI animal model

Rats were anesthetized with 4% chloral hydrate (40 mg/100 g of body weight). A midline incision (3 cm) was made in the neck to expose the common carotid vein on the right side and the common carotid artery on both sides. A tubing was inserted via the common carotid vein into the right atrium, and 500 U of heparin were infused. About 30% of the total blood volume was collected. The common carotid artery on both sides were occluded for 20 min prior to autologous transfusion of the collected blood prior to artery occlusion. The right common carotid vein was ligated and the incision was closed. The sham operation consisted of similar procedures but the common carotid arteries were not occluded [24,30,31]. Body temperature of the rats was maintained at 37.5 ± 0.5°C by the use of a heating pad during the period of ischemia and the following 2 h.

### SLM tests

SLMs were tested using a Morris water maze on the seventh day after the artery occlusion for 5 days (i.e., day 8-day 12 post-GCIRI), following a reported method [32]. The training process consisted of two steps. In step 1 (day 1), the rats were placed on the platform for 1 min, and then were made to swim freely to the platform. If the rats did not reach the platform within 3 min, they were guided to the platform manually by the researchers. On days 2–4, the training consisted of four sessions per day. A different entry site was used for each daily session, and the rats were placed underwater to search for the platform. If the rats did not reach the platform within 3 min, they were guided to the platform by the researchers and allowed to stay there for 10 sec. The maximum search time was set at 3 min. In step 2 (day 5), the platform was removed, and the rats were placed in the entry site where the last training was performed. The latency was recorded with a maximum of 3 min.

### Histopathological and IHC examinatios

Rats were anesthetized with 4% chloral hydrate (40 mg/100 g) prior to perfusion with 4% paraformaldehyde. The brains were removed and stored in 4% paraformaldehyde. Brain sections (each 5-μm) were prepared for staining with hematoxylin-eosin (HE). The morphology of hippocampal neurons and changes at the cellular level were observed.

An immunohistochemical method was used to test the expression of the NF-κB p65 protein. The sections were obtained from the same paraffin blocks as used for histological evaluation. High pressure antigen retrieval was performed in citrate buffer for 10 min prior to peroxidase quenching with 3% H_2_O_2_ in phosphate-buffered saline (PBS) for 10 min. The sections were then washed in water and preblocked with normal goat serum for 10 min. Then, slides were incubated with a rabbit polyclonal antibody raised against NF-κB p65 (1:50, Santa Cruz, CA) [24] overnight at 4°C. The sections were then incubated with biotinylated secondary antibodies (1:400, BIO-LAB, China) [33] for 20 min. Following a washing step with PBS, the avidin-biotin complex was applied. Finally, the sections were rinsed in PBS, developed with diaminoben-zidine tetrahydrochloride substrate for 3 min and counterstained with hematoxylin. The Integral Optical Density (IOD) were analyzed by image-pro plus 6.0 software.

### Reverse transcription polymerase chain reaction (RT-PCR) of PPARs mRNA

Total RNA was extracted from hippocampal tissue using TRIzol reagent (BioFlux). The RT-PCR system included 1 μg total RNA, 1 μmol/L oligo(dT), 0.2 mmol/L dNTPs, 10 U RNase inhibitor and 4 U ReverTra Ace (FSK-100, Toyobo). The reaction consisted of 20 min at 42°C, 5 min at 99°C and then 5 min at 4°C. The primers of PPARs were designed using Primer Premier 5.0 (Premier Biosoft International, Palo Alto, California, USA) on the basis of the rat PPARs cDNA sequence in genebank and synthesized by Sangon Biotech Co., Ltd. (Shanghai, China). The primers of endogenous β-Actin were purchased from DINGGUO Biotech Incorporated Company (Beijing, China). The primers were: for PPAR-α, forward (F): 5'-ACGATGCTGTCCTCCTTGATG-3', reverse (R): 5'-GCGTCTGACTCGGTCTTCTTG-3'; for PPAR-β, F: 5'-GCCGCCCTACAACGAGATCA-3'; R: 5'-CCACCAGCAGTCCGTCTTTGT-3; for PPAR-γ, F: 5'-CCCTTTACCACGGTTGATTTCTC-3', R: 5'-GCAGGCTCTACTTTGATCGCACT-3'. Primers for β-actin were purchased from Beijing Dingguo Biotechnology: F: 5'-GTGGGGCGCCCCAGGCACCA-3'; R: 5'-CTTCCTTAATGTCACGCACGATTTC-3'. Amplification was carried out in 0.2 mmol/L dNTPs, 2 mmol/L MgCl_2_, 1 μmol/L of each primer, and 2.5 U Taq DNA polymerase (Promega), and consisted of the following steps: initial denaturation at 94°C for 4 min, followed by 35 cycles of 94°C for 15 sec, 53.1°C for 15 sec and 72°C for 40 sec, annealing at 55.2–57°C (at 55.2, 57.0 and 53.1°C for PPAR-α, R-β and -γ respectively for 15 sec), and a final extension at 72°C for 5 min. The amplified products were separated with 2% agarose gel electrophoresis. The optical density of PPAR-γ was determined with Quantity One software (Bio-Rad), and expressed as the ratio against β-actin.

### PPARs western blot

Hippocampal tissue (50 mg) was homogenized in 0.5 ml tissue lysate and centrifugated at 12,000 g at 4°C for 5 min. The supernatant was (20 μg protein) was subjected to sodium dodecyl sulfate polyacrylamide gel electrophoresis (SDS-PAGE) and electrotransferred to polyvinylidene fluoride (PVDF) membranes. The membranes were blocked in 5% nonfat milk for 1 hour at room temperature. After washing in phosphate buffer solution (PBS) for 3 times, the membrane was incubated with a rabbit-anti-rat PPAR-α antibody (1:1000; Abcam, England) [30], PPAR-β antibody (1:1000; Santa Cruz, CA) [24], PPAR-γ antibody (1:1000; Abcam, England ) [30] at room temperature overnight, and then incubated with an anti-rabbit IgG conjugated with horseradish peroxidase (1:1000; Santa Cruz, CA) at 37°C for 1 hour. The color reaction was carried out using ECL reagents (Pierce, USA). A Bio-Rad imaging system was used to quantify the PPARs band.

### Biochemical analysis of hippocampal tissues

Hippocampal tissue was homogenized with physiological saline at a ratio of 1:9 (weight/volume) in a glass homogenizer. The homogenate was centrifuged at 3,000 rpm for 10 min, and the supernatant was collected to determine superoxide dismutase (SOD) activity using an SOD reagent (Nanjing Jiancheng Bioengineering Institute, China) and to determine the malondialdehyde (MDA) content using a MDA kit (Nanjing Jiancheng Bioengineering Institute). The levels of inflammatory cytokines (TNF-α, IL-1β, IL-6 and IL-10) were measured using ELISA kits (ADI, Stamford, USA).

### Statistical analysis

All data are expressed as mean ± SD, and analyzed using Statistical Package for the Social Sciences v. 12.0 (SPSS 12.0, SPSS Inc., Chicago, IL, USA). Differences among groups were analyzed by one-way analysis of variance (ANOVA), Student Newman Keuls (SNK) and SNK-q tests. Results were considered significant if the two-tailed P was <0.05.

## Results

### Effect of rosiglitazone on learning and memory

**Figure 1:**
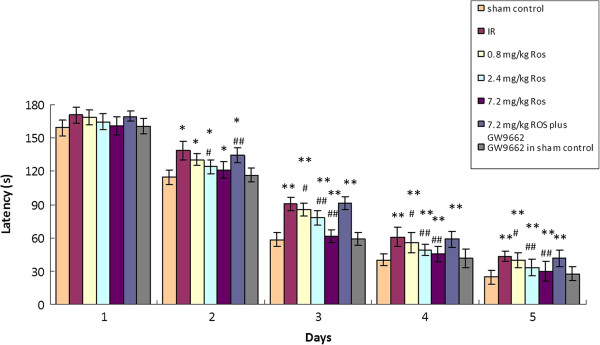
**Effects of rosiglitazone on spatial learning and memory function (n = 12).** * and **: P < 0.05 and 0.01, vs. sham control; ^#^ and ^##^: P < 0.05 and 0.01 vs. IR.

Relative to the sham controls, rats with GCIRI had significantly longer platform-seeking time during the training phase as well as markedly longer latency on the test day. Rosiglitazone dose-dependently reduced the platform-seeking time in the learning and memory phases in rats with GCIRI. GW9662 attenuated the effects of rosiglitazone, but no significant difference was observed between the sham control and the GW9662 in sham control groups (Figure 1).

### Histopathological changes

**Figure 2:**
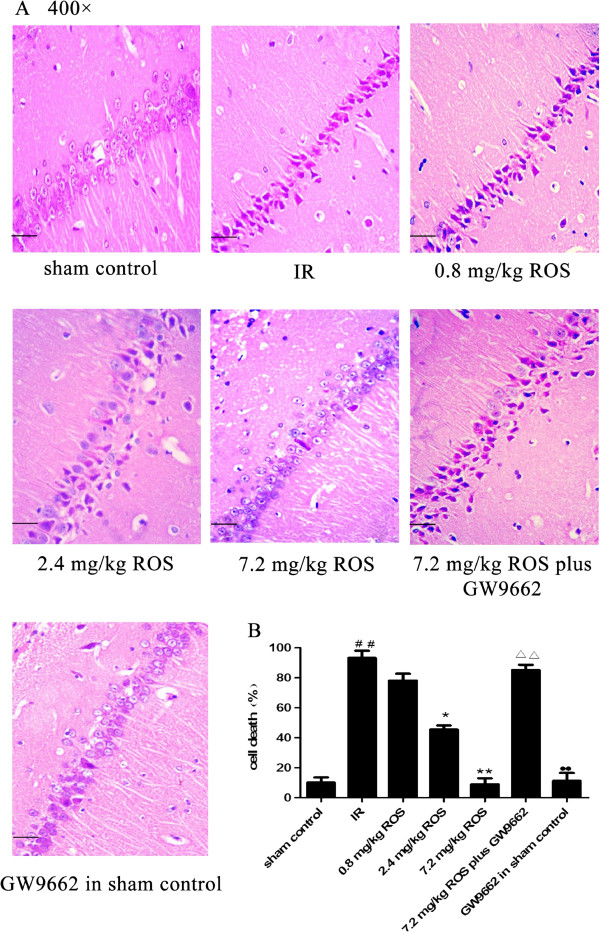
**Effects of rosiglitazone on hippocampal neurons in rats with global cerebral IRI (HE stain, ×400, n = 3). A**: Representative pictures of H&E stained CA1 sections on Day 12 post-IRI shown at 400 × magnification. Scale bars = 50 μm. **B**: Group data showing the effect of rosiglitazone on the cell death rate. ^##^P < 0.01 compared with vehicle sham group; *P < 0.05 **P < 0.01 compared with the IR group; ^ΔΔ^P < 0.05 compared with 7.2 mg/kg ROS group; ^●●^P < 0.05 compared with 7.2 mg/kg ROS plus GW9662 group.

GCIRI resulted in robust nuclear pyknosis and a reduction in the number of neurons in the hippocampus. In rats pretreated with 7.2 mg/kg rosiglitazone, hippocampal neurons were clear and intact in structure, and cells were aligned properly. Nuclear pyknosis was evident in the hippocampus of rats pretreated with 0.8 or 2.4 mg/ rosiglitazone. GW9662 attenuated effects of rosiglitazone on nuclear pyknosis, but no significant difference was found between the sham control and the GW9662 in sham control groups (Figure 2).

### Levels of IL-1β, IL-6, IL-10 and TNF-α

GCIRI increased the levels of inflammatory cytokines IL-1β, IL-6 and TNF-α in the hippocampus. The IL-10 level was markedly decreased. Rosiglitazone, in a dose-dependent manner, attenuated I/R-induced elevation of TNF-α, IL-1β and IL-6 levels and the reduction of IL-10. The effects of rosiglitazone were inhibited by GW9662. No significant difference was observed between the sham control and the GW9662 in sham control groups (Table 1).

**Table 1:** Effects of rosiglitozane on IL-1b,IL-6,IL-10 and TNF-a content (n = 5)

**Group**	**IL-1β**	**IL-6**	**IL-10**	**TNF-α**
	**(mg/ml)**	**(ng/ml)**	**(ng/ml)**	**(ng/ml)**
sham control	218.35 ± 42.2	6.37 ± 0.43	3.01 ± 0.25	2.19 ± 0.35
IR	561.31 ± 49.98**	15.83 ± 1.37*	1.86 ± 0.15**	6.36 ± 1.11**
0.8 mg/kg Ros	505.11 ± 39.28**	13.27 ± 0.89**	2.81 ± 0.37**	5.57 ± 0.43**
2.4 mg/kg Ros	437.71 ± 51.74^**##^	10.27 ± 0.516^**##^	2.37 ± 0.25^**##^	4.23 ± 0.61^**##^
7.2 mg/kg Ros	390.22 ± 42.08^##^	8.05 ± 0.95^##^	2.01 ± 0.31^##^	3.39 ± 0.37^##^
7.2 mg/kg Ros plus GW9662	572.40 ± 33.27**	16.17 ± 1.12**	1.95 ± 0.17**	6.19 ± 1.01**
GW9662 in sham control	233.57 ± 28.33	6.07 ± 0.33	3.21 ± 0.35	2.45 ± 0.22

Datas are expressed as mean ± SD (n = 5). * and **:P < 0.05 and 0.01, vs. sham control; ^#^ and ^##^:P < 0.05 and 0.01 vs. IR.

### MDA levels and SOD activity

Global cerebral I/R resulted in significant increases of MDA content and a decrease in SOD activity in the hippocampus compared to the sham control group. Rosiglitazone attenuated I/R-induced elevation of MDA levels and reduction of SOD activity. The effects of rosiglitazone were inhibited by GW9662, but there was no significant difference between the sham control and the GW9662 in sham control groups (Table 2).

**Table 2:** Effects of rosiglitozane on MDA content and SOD activity (n = 5)

**Group**	**SOD**	**MDA**
	**(ng/ml)**	**(ng/ml)**
sham control	71.58 ± 6.23	1.74 ± 0.14
IR	37.24 ± 5.16**	2.33 ± 0.19**
0.8 mg/kg Ros	40.37 ± 4.32**	2.14 ± 0.15**
2.4 mg/kg Ros	49.37 ± 7.25^**##^	1.97 ± 0.17*^##^
7.2 mg/kg Ros	55.47 ± 9.31^##^	1.89 ± 0.21^##^
7.2 mg/kg Ros plus GW9662	35.31 ± 6.11**	2.44 ± 0.17**
GW9662 in sham control	69.43 ± 4.57	1.83 ± 0.24

Datas are expressed as mean ± SD (n = 5). * and **:P < 0.05 and 0.01, vs. sham control; ^#^ and ^##^:P < 0.05 and 0.01 vs. IR.

### NF-κB p65 expression

**Figure 3:**
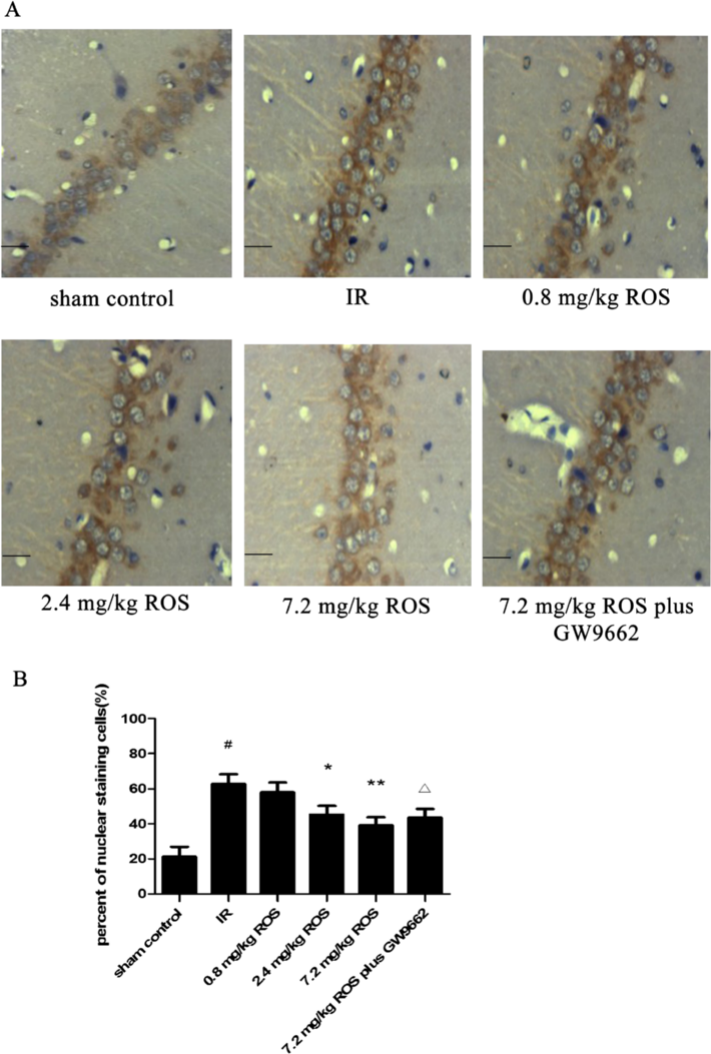
**Effects of rosiglitazone on NF-κB expression in hippocampal tissues of rats with global cerebral IRI (HE stain, ×400, n = 3). A**: representative images of the hippocampal CA1 region after IRI. (Scale bars = 50 μm). **B**: Group data showing the effect of rosiglitazone on NFκB expression and translocation. ^#^P < 0.05 compared with sham control group; *P < 0.05 **P < 0.01 compared with the IR group; ^Δ^P < 0.05 compared with 7.2 mg/kg ROS group.

IHC results revealed a significant increase of NF-κB p65 expression in hippocampal neuronal cytoplasm. Rosiglitazone decreased the expression of NF-κB p65 protein in rats subjected to GCIRI. Such an effect was attenuated by GW9662, but there was no significant difference between the sham control and the GW9662 in sham control groups (Figure 3).

### PPAR-α, PPAR-β and PPAR-γ mRNA

**Figure 4:**
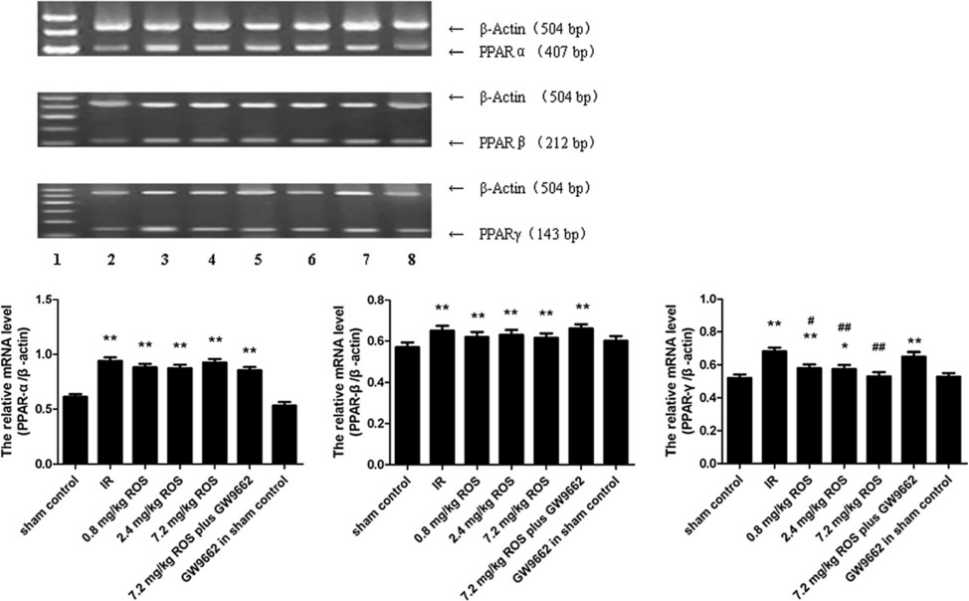
**Effects of rosiglitazone on expression of PPARs (A, PPAR-α; B, PPAR-β; C, PPAR-γ) mRNA in hippocampus of rats with global cerebral IRI.** Lane 1, sham control; Lane 2, IR ; Lane 3, 0.8 mg/kg ROS; Lane 4, 2.4 mg/kg ROS; Lane 5, 7.2 mg/kg ROS; Lane 6, 7.2 mg/kg ROS plus GW9662; Lane 7, GW9662 in sham control. *and **P < 0.05 and 0.01, VS sham control, ^#^ and ^##^P < 0.05 and 0.01, VS IS group. (n = 4).

GCIRI resulted in significant increases in PPAR-α, PPAR-β and PPAR-γ mRNAs compared with that of the model control group (p < 0.01). Rosiglitazone at 0.8, 2.4 and 7.2 mg/kg inhibited the increase of the PPAR-γ mRNA expression (p < 0.05, <0.01 and <0.01, respectively), with an inhibition rate of 10.79%, 20.63% and 32.33%, respectively, in a dose-dependent manner. PPAR-α and PPAR-β were not affected. GW9662 had no significant effect on the expression of PPARs in the sham control group (Figure 4).

### PPAR-α, PPAR-β and PPAR-γ proteins

**Figure 5:**
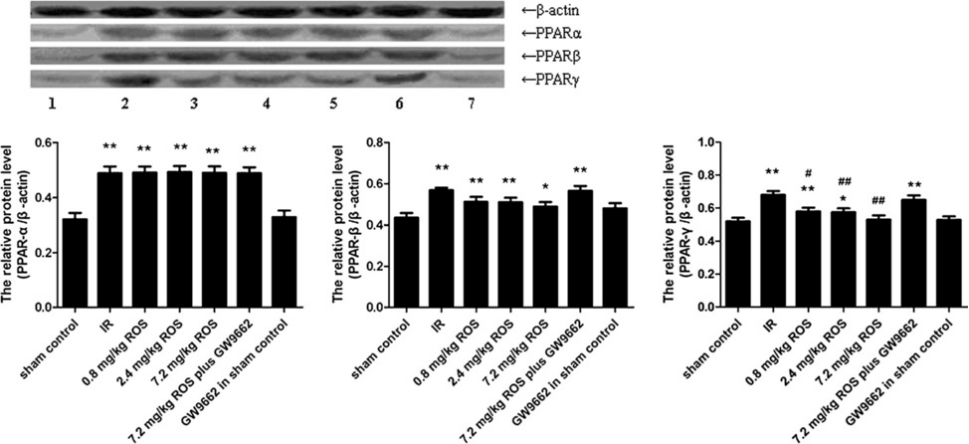
**Effects of rosiglitazone on expression of PPARs (A, PPAR-α; B, PPAR-β; C, PPAR-γ) proteins in hippocampus of rats with global cerebral IRI.** Lane 1, sham control; Lane 2, IR ; Lane 3, 0.8 mg/kg ROS; Lane 4, 2.4 mg/kg ROS; Lane 5, 7.2 mg/kg ROS; Lane 6, 7.2 mg/kg ROS plus GW9662; Lane 7, GW9662 in sham control. *and **P < 0.05 and 0.01, VS sham control, ^#^ and ^##^P < 0.05 and 0.01, VS IR group. (n = 4).

GCIRI resulted in significant increases in the protein levels of PPAR-α, PPAR-β and PPAR-γ. Rosiglitazone decreased the PPAR-γ protein in the hippocampus of rats with GCIRI in a dose-dependent way. PPAR-α and PPAR-β were not affected, and there was no significant difference between the sham control and the GW9662 in sham control groups (Figure 5).

## Discussion

GCIRI occurs in patients who are successfully resuscitated from various clinical conditions such as cardiac arrest, asphyxia and shock, because of the limited therapeutic window (2–3 h after onset of the symptoms), but very few patients with cardiac arrest receive timely and effective treatment. Therefore, it is of great importance to investigate the molecular mechanisms of such injury caused by ischemic cerebrovascular disease, and thereby to develop effective drugs for treatment.

Our study showed that GCIRI induced inflammation and oxidative stress in the hippocampus of rats. In the hippocampus of rats undergoing GCIRI, the expression of PPARs and the level of NF-κB increased. Rosiglitazone attenuated the damage of hippocampal neurons and improved SLM functions. Such effects were attenuated by the PPAR-γ antagonist GW9662. Our results suggest that PPAR-γ agonists produce anti-inflammatory action by inhibiting the activation of the NF-κB signaling pathway and the expressions of inflammatory factors. Intriguingly, neither the agonist nor the antagonist of PPAR-γ had any effect on the expression of PPAR-α and PPAR-β.

Recent reports show that PPARs have a neuroprotective effect on the CNS. Fibrates as PPAR-α ligands have antioxidant and anti-inflammatory effects on GCIRI of female rats, and pretreatment with gemfibrozil prior to I/R can modulate inflammatory factors and stimulate the antioxidant defense system [34]. In an experimental model of spinal cord injury in mice, simvastatin can inhibit the severity or level of spinal cord inflammation, neutrophil infiltration, pro-inflammmatory cytokine expression and apoptosis [35]. In addition, PPAR-β has become a focus in the field of neuronal damage. Compared with the wild type, the PPAR-β-null mice exhibit a significantly larger size of infarct in a model of focal cerebral ischemia established by MCAO [36,37]. Preactivation of PPAR-β could dose-dependently improve SLM function and cytomorphological change of the hippocampal neurons in the rats with GCIRI [24].

In a previous study on rats, intraventricular administration of pioglitazone at 1 h before and 24 h after MCAO separately reduced the infarct area and improved neurological function [38]. Another study demonstrated that in cultured astrocytes and microglia, the expressions of lipopolysaccharide (LPS)-induced IL-6, TNF-α, iNOS, and COX-2 were elevated. The PPAR-γ agonists thiadiazolidinones up-regulate the expression of LPS-induced iNOS, and reduce the productions of NO, IL-6 and TNF-α in a culture. The neuroprotective effects of thiadiazolidinones were abolished by GW9662 [39,40]. Treatment with PPAR-γ agonists before MCAO decreases the expressions of ROS and iNOS, decreases lipid peroxidation and reverses glutathione (GSH) exhaustion in the hippocampus [41]. Pioglitazone inhibits the LPS-induced expression of COX-2 in the primary cortical neurons and prevents neuronal apoptosis induced by oxidative stress [42]. A more recent investigation of the effects of PPAR-γ agonists on the NF-κB signaling pathway [43]showed that upon MCAO, the p65 subunit of NF-κB was transferred from the cytoplasm to the nucleus of the hippocampal neurons. Treatment with pioglitazone subdued the transferrence of the NF-κB p65 subunit to the nucleus. These findings are consistent with our observations and indicate that PPAR-γ agonists are neuroprotective against GCIRI.

The above results indicate that PPAR-α, -β and especially PPAR-γ may be important targets for GCIRI research and drug therapy. Although many studies have reported I/R can induce the increased PPAR-γ expression [44,45], it is unknown whether the inhibition or activation of the PPAR-γ has any effect on the expression of PPAR-α and -β. This study shows that the expression of PPAR-γ in the hippocampus of GCIRI rats increased, and the expressions of PPAR-α and -β also increased. Moreover, agonists and antagonists given to PPAR-γ had no obvious effect on the expression of PPAR-α or -β, suggesting that PPAR-α, -β and -γ act in separate pathways. The intervention of GCIRI by changing the activity or expression of PPAR-γ resulted in minimal side effects from the PPAR-α and -β expression. Moreover, since PPAR-α, -β and -γ were three independent pathways, and since the activation or expression of all of them could protect neurons by reducing neuronal inflammation and oxidative stress injury, a superimposed effect may be produced if the three are activated or expressed at the same time. Such hypotheses will be verified in the following experiment to provide new ideas and experimental basis for combined therapy for cerebral ischemic injury by using PPAR-α, -β and -γ agonists.

## Conclusions

The present study demonstrated that the activation or antagonism of PPAR-γ has no effect on the expression of PPAR-α or -β in the hippocampus of rats with GCIRI, and the three PPAR isotypes act in three independent pathways in the CNS.

## Abbreviations

Aβ: Amyloid-β; CNS: Central nervous system; CuZn-SOD: CuZn- superoxide dismutase; DBD: DNA binding domain; DEPC: Diethylpyrocarbonate; EB: Ethidium bromide; ELISA: Enzyme-linked immunosorbent assay; GCIRI: Global cerebral ischemia/reperfusion injury; HE: Hematoxilin and Eosin; IL-1β: Interleukin-1β; IL-6: Interleukin-6; IL-10: Interleukin-10; iNOS: Inductible nitric-oxide synthase; LBD: ligand-binding domain; LPS: lipopolysaccharide; MCAO: Middle cerebral artery occlusion; MDA: Malondialdehyde; MPTP: 1-methyl-4 -phenyl-1,2,3,6-tetrahyropyridine; NF-κB: Nuclear factor-κB; mm: Millimeter; NO: Nitric oxide; PBS: Phosphlate-buffered saline; PEA: Palmitoylethanolamide; PPARs: Peroxisome proliferator-activated receptors; PPRE: Peroxisomal proliferator response element; RT-PCR: Reverse transcription polymerase chain reaction; SD: Sprague–Dawley; SML: Spatial learning and memory; SOD: Superoxide dismutase; Taq: Taq DNA; TE: Tris-EDTA buffer; TNF-α: Tumor necrosis factor-α; WB: Western blotting.

## Competing interest

The authors declare that they have no competing interest.

## Authors’ contributions

Ying collected the data, analyzed and interpreted data, drafted the manuscript. Junqing conceived the idea, designed the study, and participated in writing up and revising the manuscript. Ge, Qin and Qingsong collected the data and assisted data analysis and paper writing. All authors read and approved the final manuscript.
